# The psychopathological trajectories to delusion in Schizophrenia: the affective and schizotypal pathways

**DOI:** 10.1192/j.eurpsy.2022.1968

**Published:** 2022-09-01

**Authors:** D. Borrelli, R. Ottoni, S. Maffei, N. Fascendini, C. Marchesi, M. Tonna

**Affiliations:** 1 University of Parma, Unit Of Neuroscience, Psychiatric Unit, Parma, Italy; 2 University of Parma, Department Of Medicine & Surgery, Unit Of Neuroscience, Psychiatric Unit, Parma, Italy; 3 Local Health Service, Department Of Mental Health, Parma, Italy

**Keywords:** delusion, Psychosis, Psychopathology, schizophrénia

## Abstract

**Introduction:**

Delusions are a key feature of schizophrenia psychopathology. From a phenomenological approach, Jaspers (1913) differentiates between “primary” or true schizophrenic delusions, defined as an unmediated phenomenon that cannot be understood in terms of prior psychological origin or motivation, and “secondary” delusions, understandable from the patient’s mood state or personality. Primary delusions have been considered the hallmark of reality distorsion dimension in schizophrenia, disregarding a possible affective patwhay to delusional belief.

**Objectives:**

The present study was aimed at elucidating the psychopathological trajectories to delusion in schizophrenia through the investigation of both affective and schizotypal trait dispositions.

**Methods:**

Seventy-eight participants affected by schizophrenia were administered the Peters Delusional Inventory (PDI), the Positive and Negative Affective Scale (PANAS), the Experience of Shame Scale (ESS), the Referential Thinking Scale (REF), the Magical Ideation Scale (MIS) and the Perceptual Aberration Scale (PAS).

**Results:**

The severity of delusional ideation (PDI) was positively related to both affective (PANAS positive dimension, ESS) and schizotypal traits (MIS, PAS and REF). Moreover, referential thinking (REF) mediated the relationship between “magical ideation” (MIS) and delusions severity (Fig. 1), whereas experience of shame (ESS) was a moderating factor in the between referential thinking and delusion severity (Fig. 2).

**Figure:**
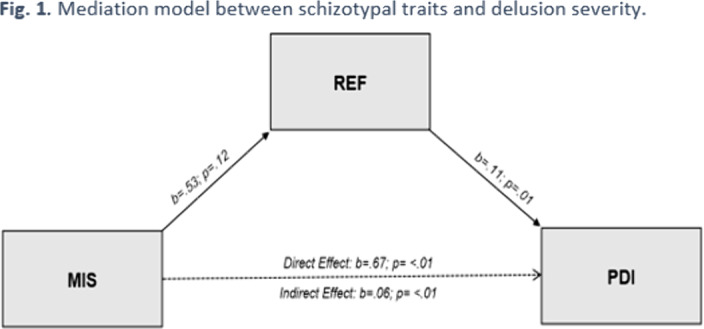

**Figure:**
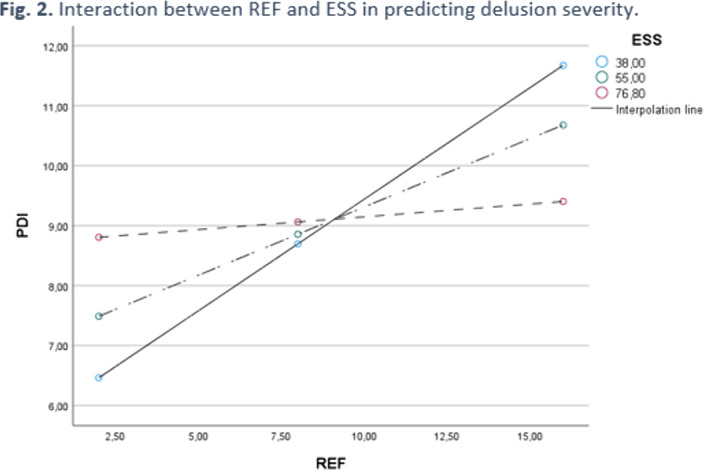

**Conclusions:**

The study findings suggest that in schizophrenia patients, severity of delusions is underpinned by an intertwining of both affective and schizotypal pathways.

**Disclosure:**

No significant relationships.

